# Book Reviews

**Published:** 1799-08

**Authors:** 


### THE VETERINARY ART.

*Tableaux comparatifs de l'Anatomie, &c.—Comparative Tables of the Anatomy of the domestic animals used in Agriculture, as Horses, Asses, Mules, Black Cattle, Sheep, Goats, Hogs, Dogs, and Cats ; arranged according to a systematic classification, with a view to facilitate that branch of study to beginners.* By J. GIRARD, Professor of Anatomy in the Veterinary School of Alfort. *Paris,* Huzard.

The author begins with pointing out the different branches of science which are necessary accomplishments to a veterinary practitioner : of these anatomy is the most important, and ought to be diligently studied, as far as it relates to the physical organization of all useful animals. He then proceeds to examine the several organic parts of the different mammalia, enumerated in the title, and divides them into four orders which he terms *monodactyles, bidactyles, regular quadridactyles,* and *irregular quadridactyles.*— The whole is arranged in a perspicuous and accurate manner, so that it may be considered a valuable elementary treatise.

*Observation sur un écoulement spermatique involuntaire dans un cheval.—On a case of an involuntary spermatic running in horses, with inquiries and remarks on the authors who have written on that disorder.* By C. HUZARD. 8vo. *Paris.*

The author gives here a distinct account of this debilitating disease in that noble animal, the horse ; together with the observations and remedies suggested by other writers on this subject ; he differs from them principally by employing caustics, where all others recommend astringents and corroborants.

